# Effect of Seafood (Gizzard Shad) Supplementation on the Chemical Composition and Microbial Dynamics of Radish Kimchi during Fermentation

**DOI:** 10.1038/s41598-019-54318-4

**Published:** 2019-11-27

**Authors:** Mohamed Mannaa, Young-Su Seo, Inmyoung Park

**Affiliations:** 1 Department of Integrated Biological Science, Pusan National University, Busan, 46241 Korea; 2 Department of Plant Pathology, Cairo University, Giza, 12613 Egypt; 3 Department of Oriental Food and Culinary Arts, Youngsan University, Busan, 48015 Korea

**Keywords:** Microbiology, Food microbiology

## Abstract

This study investigated the impact of supplementing radish kimchi with slices of gizzard shad, *Konosirus punctatus* (boneless - BLGS, or whole - WGS) on the kimchi’s chemical and microbial composition for different fermentation durations. Higher levels of amino nitrogen (N), calcium (Ca) and phosphorus (P) were observed in the supplemented kimchi groups compared to those in the control and further, Ca and P levels were highest in the WGS kimchi group. Microbial composition analysis revealed noticeable differences between the three groups at different fermentation durations. The predominant species changed from *Leuconostoc rapi* to *Lactobacillus sakei* at the optimal- and over-ripening stages in the control kimchi group. The predominant species in the BLGS kimchi group was *L. rapi* at all stages of fermentation, whereas the predominant species in the WGS kimchi group was *L. rapi* at the optimal-ripening stage, and both *L. sakei* and *L. rapi* at the over-ripening stage. Significant correlations were observed by analysis of the Spearman’s rank between and within the chemical and microbial composition over fermentation durations. Altogether, gizzard shad supplementation may be used to optimize the desired microbial population to obtain the preferable fresh kimchi flavour by the release of certain inorganic elements and amino N.

## Introduction

Since ancient times, humans have known that fermented foods and drinks are characterized by extended shelf lives and improved organoleptic properties. During the fermentation process, the microbes in the fermented food transform the substrates into bioactive, functional, and nutritious compounds. Recently, fermented foods have gained popularity for their health benefits^1,2^. Kimchi is a traditional Korean fermented food that is a basic component of the Korean daily diet. Globally, kimchi is known for its health-promoting and potential anticancer properties^3^. During the initial stages of fermentation, kimchi comprises diverse microbes that originate from various ingredients, such as vegetables (cabbage and radish), seasoning agents (salt, sugar, red pepper powder, garlic, ginger, and fermented fish source), and seafood, depending on the local preference. During fermentation, lactic acid bacteria (LAB) produce lactic acid, which drastically reduces the pH of the fermentation medium and consequently inhibits the growth of most other bacterial groups. Hence, LAB form the predominant microbial component of kimchi^4^. During fermentation, the microbes in kimchi produce various metabolites, such as functional organic acids (*e.g*. lactic and propionic acid), and flavouring (*e.g*. acetoin and 2,3-butanediol) and antioxidant (*e.g*. mannitol and the non-protein amino acid L-Ornithine) compounds, which determine the quality and properties of the fermented kimchi^5–7^.

Therefore, it is essential to examine the microbial composition of kimchi to understand the factors that affect the microbial communities during the fermentation process^8^. The microbial composition can be determined by traditional culture-based methods; however, these methods do not accurately and reproducibly depict the microbial composition. Recently, metagenomic analysis has been widely used to profile the microbial composition during kimchi fermentation, as it can overcome the limitations associated with traditional culture-based approaches^9,10^.

The factors that affect kimchi fermentation and its microbial composition have been extensively studied. The microbes involved in the fermentation of kimchi and other related fermented foods, such as sauerkraut, mainly belong to the *Leuconostoc*, *Lactobacillus*, and *Weissella* genera^11^. Generally, the predominant species during the initial stages of kimchi fermentation is *Leuconostoc mesenteroides*, which provides a fresh flavour. The predominant LAB during the optimum-ripening stage and over-ripening stage of fermentation are *Lactobacillus sakei* and *Weissella koreensis*. These LAB promote acidic deterioration and can negatively impact the fresh flavour^12–14^.

The ingredients used in kimchi preparation are not only a microbial source, but also affect the fermentation conditions, such as salt concentration, inorganic elements content, and protein amount. Additionally, some ingredients, such as garlic, have a potential intrinsic antimicrobial effect against certain species. Hence, the fermentation process depends on the ingredients used for kimchi preparation^8^ and evaluating the effect of these different ingredients on the kimchi microbiome is important to understand the fermentation process. Traditionally, different types of fresh seafood are used as ingredients for kimchi preparations in many Korean coastal provinces to improve the flavour and nutrient content, such as inorganic elements and amino acids. However, only a few types of whole fish can be used, such as anchovy, skate ray and gizzard shad, because their bones are relatively small and soft. A few studies have reported improved physicochemical properties upon addition of seafood to kimchi preparations^15,16^. Unfortunately, little is known about the effect of adding fresh seafood on the microbial composition of kimchi and its dynamics. The aim of this study was to investigate the effect of adding fresh seafood, in the form of sliced boneless (BLGS) or whole gizzard shad (WGS) fish, *Konosirus punctatus*, on the microbial dynamics of radish kimchi over different fermentation periods: initiation stage (week 0), optimal-ripening stage (week 2), and over-ripening stage (week 6) as was determined in a previous study^17^. Additionally, the effect of BLGS and WGS supplementation on the inorganic elements and amino nitrogen (N) content, and the correlation between the content and the microbial dynamics, was investigated.

## Results

### Changes in the inorganic elements and amino N contents in kimchi supplemented with BLGS or WGS over different fermentation periods

**Figure 1:**
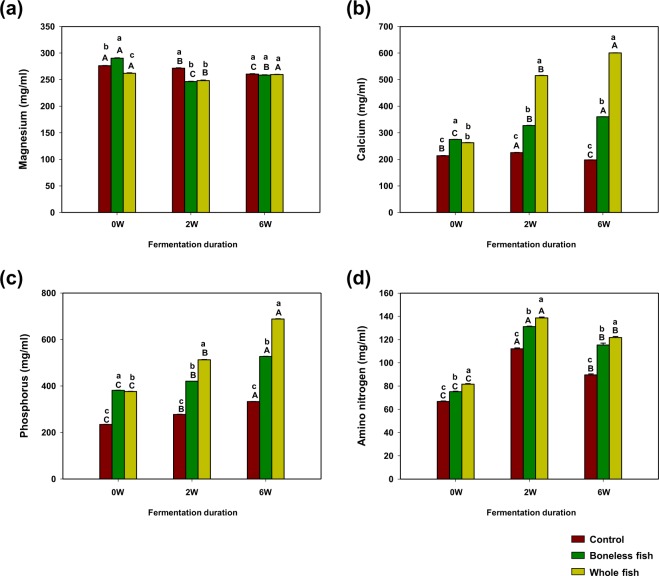
Quantification of different inorganic elements [(**a**) magnesium, Mg; (**b**) calcium, Ca; (**c**) phosphorus, P and (**d**) amino nitrogen], in radish kimchi prepared by adding 10% boneless or whole gizzard shad fish slices. The control kimchi group was prepared without adding gizzard shad fish. The different lowercase and uppercase letters on the error bar indicate significant difference (*P* < 0.05) between different kimchi groups at a given fermentation duration and between the different fermentation durations within each kimchi group, respectively.

Significant differences (*P* < 0.05) were observed in the inorganic elements contents [magnesium (Mg), calcium (Ca), and phosphorus (P)] among the control, BLGS- and WGS-supplemented groups over various fermentation periods (Fig. 1a–d). A slight reduction in the Mg content was observed as fermentation progressed. The Mg content at the optimal-ripening stage in the control kimchi group was significantly (*P* < 0.05) higher than that in the other two kimchi groups (Fig. 1a). The Ca, and P contents increased significantly (*P* < 0.05) as fermentation progressed in the BLGS and WGS kimchi groups. The Ca and P contents in the WGS kimchi group were markedly higher than those in the other two kimchi groups (Fig. 1b,c). The amino N content in the WGS kimchi group was higher than that in the BLGS kimchi group throughout the fermentation period. The amino N content increased at the optimal-ripening stage and decreased at the over-ripening stage of fermentation (Fig. 1d).

### Changes in microbial diversity and richness over fermentation duration

**Figure 2:**
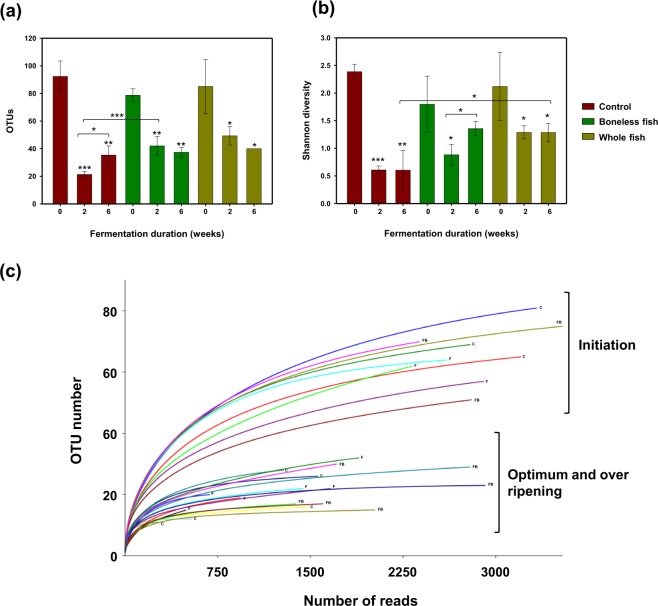
Alpha diversity and rarefaction curves of the microbiota of radish kimchi prepared by adding 10% boneless or whole gizzard shad fish slices over different fermentation durations. The control kimchi group was prepared without adding gizzard shad fish. (**a**) Number of operational taxonomic unites (OTUs), (**b**) Shannon richness index. Data are presented as the mean ± standard deviation for each kimchi group. ***P < 0.001, **P < 0.01, and *P < 0.05. (**c**) Rarefaction curve of the 16S rRNA sequence reads against the assigned operational taxonomic units (OTUs) to evaluate whether further sequencing may result in detecting additional taxa.

The richness and diversity of the microbiome in all three radish kimchi groups at the initiation stage of fermentation were significantly (*P* < 0.05) higher than those at the optimal- and over-ripening stages of fermentation, as revealed by the total number of operational taxonomic units (OTUs) and the Shannon diversity index (Fig. 2a,b). A similar trend was observed in the other alpha diversity and richness measurements (Chao1 richness and inverse Simpson indices) (Fig. S1). The Shannon diversity index revealed that the microbial diversity in the WGS kimchi group was higher than that in the control kimchi group, particularly at the over-ripening stage. Additionally, the microbial diversity at the over-ripening stage was higher than that at the optimal-ripening stage in the BLGS kimchi group (Fig. 2b). The rarefaction curves at 97% similarity demonstrated a good coverage of the sequencing depth, with levelling of the curves by approximately 1000 reads for samples at the initiation of fermentation. The decrease in the number of OTUs with the progression of fermentation was inferable from the rarefaction curves, as was also revealed by the alpha diversity and richness measurements (Fig. 2c).

**Figure 3:**
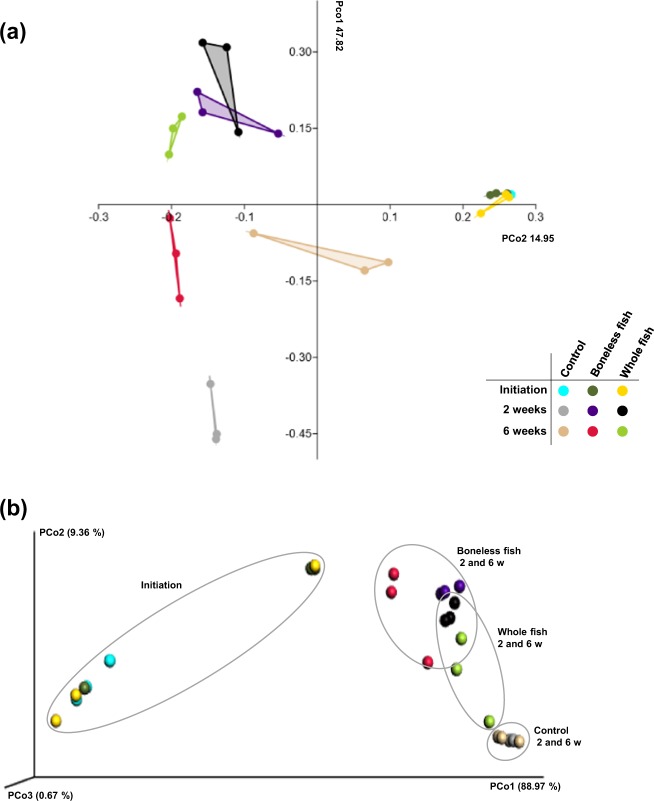
Principle coordinate analysis (PCoA) representing the beta-diversity in the microbiota of the three radish kimchi groups. The PCoA was estimated based on **(a**) Bray-Curtis, and (**b**) weighted UniFrac distances. Each dot represents a replicate of the different groups of kimchi prepared by adding 10% boneless or whole gizzard shad fish slices over different fermentation durations (initiation, 0 weeks; optimal ripening, 2 weeks; over ripening, 6 weeks). The control kimchi group was prepared without adding gizzard shad fish. Clustering was clear between the kimchi groups at the initial stage of the fermentation and at the optimal-ripening (2 weeks) and the over-ripening stages (6 weeks).

For the beta-diversity assessment, the similarity in the microbial communities between the three radish kimchi groups over the fermentation duration was plotted as principal coordinates analysis (PCoA) using the Bray-Curtis and weighted UniFrac distances. The distance (Bray-Curtis and weighted UniFrac) measurements revealed a strong clustering between the three kimchi groups at the initiation stage of fermentation. Clustering was also observed between the three kimchi groups at the optimum- and over-ripening stages, particularly within the control kimchi group. The clustering of the microbiome in the control kimchi group was more intact compared with that in the other two kimchi groups (Fig. 3a,b). Similar results were obtained when the beta diversity was measured using the Euclidean distance (Fig. S2). These results indicate that supplementing with BLGS or WGS during fermentation affects the microbial composition of kimchi. Additionally, the effect of fermentation time was more drastic in the BLGS and WGS kimchi groups than in the control kimchi group between the initiation stage, and optimal- and over-ripening stages (Fig. 3a,b).

### Microbiome composition in different kimchi groups with different fermentation durations

**Figure 4:**
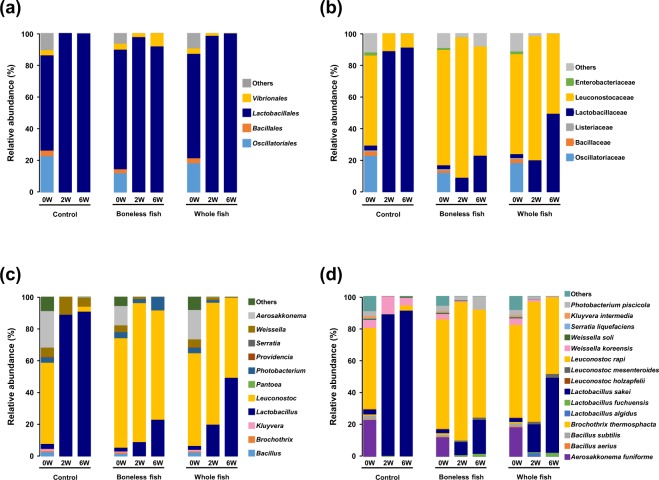
Stacked bar graphs representing the microbiome structure of radish kimchi prepared by adding 10% boneless or whole gizzard shad fish slices over different fermentation durations. The control kimchi group was prepared without adding gizzard shad fish. Microbial composition represented at the (**a**) order level, (**b**) family level, (**c**) genus level, showing the eleven most abundant genera, and (**d**) species level, showing the fifteen most abundant species.

Noticeable changes were observed in the relative abundance of different bacterial taxa among the three kimchi groups at the initiation, optimal- and over-ripening stages of fermentation. At the initiation stage, the Lactobacillales order was the predominant microbial community among the three kimchi groups (Fig. 4a). There was no difference in the microbial composition from the phylum to the order level between the BLGS or WGS kimchi groups and the control kimchi group (Figs. 4a and S3). However, the microbial community showed a notable difference at the family level between the control kimchi group and the BLGS and WGS kimchi groups. Lactobacillaceae was the predominant family in the control kimchi group, whereas Leuconostocaceae was the predominant family in the BLGS and WGS kimchi groups. At the over-ripening stage of fermentation, both Lactobacillaceae and Leuconostocaceae families were equally predominant in the WGS kimchi group (Fig. 4b).

The microbial compositions showed a similar distinction between the three kimchi groups at the genus and species levels. *Lactobacillus* was the predominant genus in the control kimchi group, whereas *Leuconostoc* was the predominant genus in the BLGS and WGS groups at the optimal-ripening stage. However, both the genera were equally predominant in the WGS kimchi group at the over-ripening stage (Fig. 4c). The predominant species in the control kimchi group was *Lactobacillus sakei* with a relative abundance of approximately 90% at the optimal- and over-ripening stages. The predominant species in the BLGS kimchi group was *Leuconostoc rapi* with a relative abundance of 86% at the optimal-ripening stage and 67% at the over-ripening stage. The predominant species in the WGS kimchi group was *L. rapi* with a relative abundance of 75% at the optimal-stage and 48% at the over-ripening stage. The second-most predominant species in the BLGS and WGS kimchi groups was *L. sakei* (Fig. 4d).

**Figure 5:**
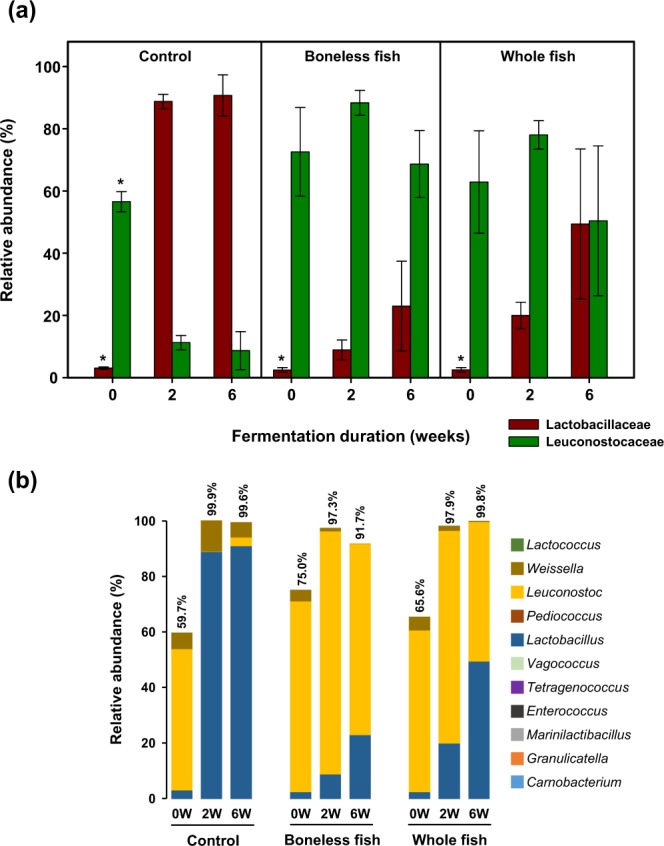
Changes in the abundance and structure of lactic acid bacteria (LAB) in radish kimchi prepared by adding 10% boneless or whole gizzard shad fish slices over different fermentation durations. The control kimchi group was prepared without adding gizzard shad fish. (**a**) The difference in the abundance of the two main LAB families. An asterisk (*) on the error bar represent statistical significance (*P* < 0.05) in the abundance of Lactobacillaceae and Leuconostocaceae over fermentation durations. (**b**) Stacked bar graph showing abundance of the Lactobacillales order and the relative abundance among the LAB genera within.

The relative abundance of the two main families in the Lactobacillales order, Lactobacillaceae and Leuconostocaceae, in the three kimchi groups over different fermentation durations was analysed. The analysis indicated that at the initiation stage of fermentation, Leuconostocaceae was the predominant family in all the three kimchi groups. Additionally, the abundance of Lactobacillaceae was significantly (*P* < 0.05) lower compared with that of Leuconostocaceae. Furthermore, the abundance of Leuconostocaceae was significantly (*P* < 0.05) reduced, whereas that of Lactobacillaceae was significantly (*P* < 0.05) increased in the control group compared with those in the BLGS or WGS groups at the optimal- and over-ripening stages (Fig. 5a). No significant difference was observed in the abundance of Leuconostocaceae in the BLGS or WGS kimchi groups over different fermentation periods. However, the abundance of Lactobacillaceae slightly increased at the optimal- and over-ripening stages of fermentation compared with that at the initiation stage (Fig. 5a). The three main genera observed within the Lactobacillales order were *Lactobacillus*, *Leuconostoc*, and *Weissella* (Fig. 5b). The predominant genus in the control kimchi group was *Lactobacillus*, which accounted for about 90% of the Lactobacillales order, followed by *Weissella* with a relative abundance of 11% and 5% at the optimal- and over-ripening stages of fermentation, respectively. The two main genera in the BLGS and WGS kimchi groups were *Leuconostoc* and *Lactobacillus*. Low abundance of *Weissella* was also observed, which further decreased over the fermentation period in the BLGS and WGS kimchi groups (Fig. 5b).

**Figure 6:**
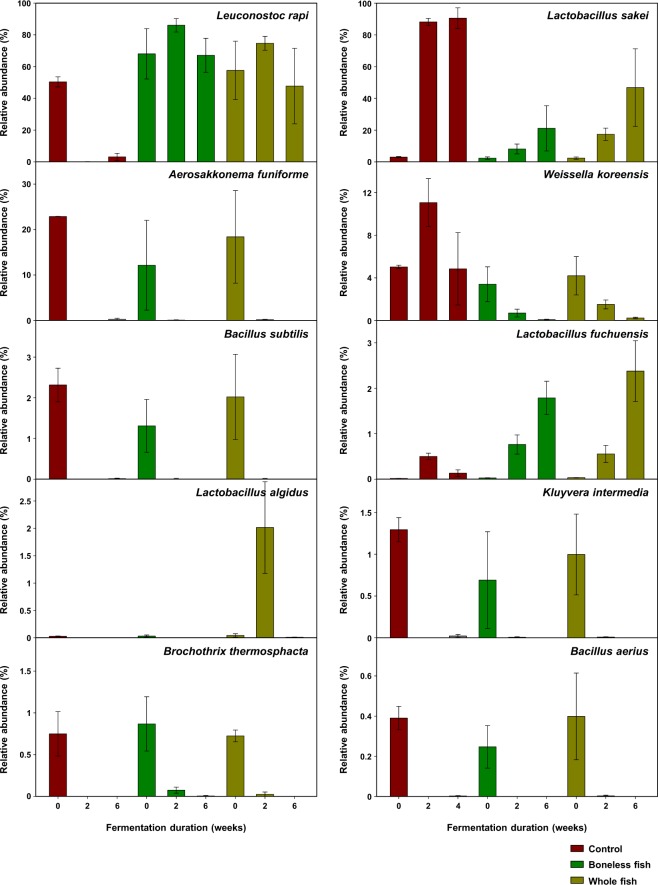
Relative abundance of ten most prevalent bacterial species in the microbiome of radish kimchi prepared by adding 10% boneless or whole gizzard shad fish slices over different fermentation durations. The control kimchi group was prepared without adding gizzard shad fish. The bars represent the mean ± standard deviation.

The comparison of the relative abundance of the ten most prevalent species in the three kimchi groups over different fermentation periods is shown in Fig. 6. The BLGS and WGS kimchi groups exhibited a higher abundance of *L. rapi* and *Lactobacillus fuchuensis* and a lower abundance of *L. sakei* and *Weissella koreensis* compared with the control kimchi group. No difference in the abundance of bacterial species was observed between the BLGS and WGS kimchi groups, except at the over-ripening stage where a higher abundance of *Lactobacillus algidus* was observed in the WGS kimchi group compared with that in the BLGS kimchi group (Fig. 6).

### Correlation between different bacterial taxa, inorganic elements and amino N contents, and fermentation duration in different radish kimchi groups

**Figure 7:**
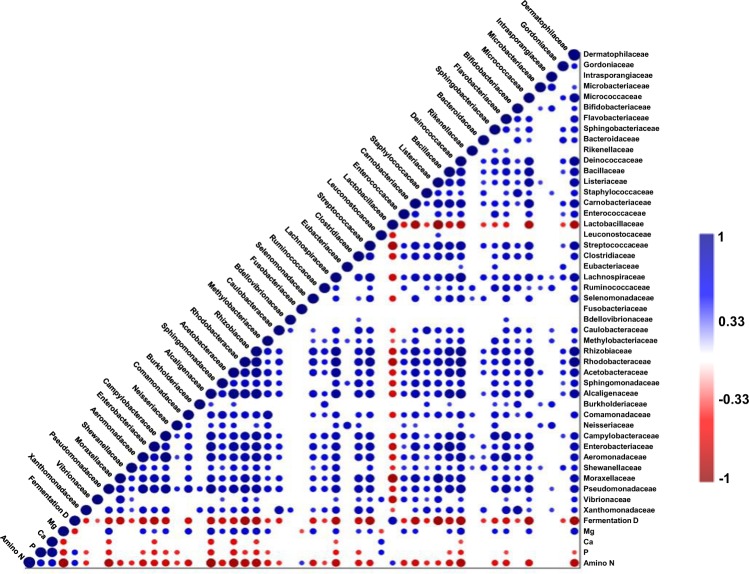
Correlogram representing the matrices of Spearman’s rank order correlation coefficient (r) between the different bacterial taxa and the fermentation duration, the magnesium (Mg), calcium (Ca), phosphorus (P) inorganic minerals and the amino nitrogen (N) contents of all three radish kimchi samples (*n* = 27) at the family levels. Only significant (*P* < 0.05) positive (blue) and negative (red) correlations are shown in the graph.

The correlograms shown in Fig. 7 represent the matrices of the Spearman’s rank order correlation coefficient (r) between different bacterial taxa at the family level, and the fermentation duration, inorganic element (Mg, Ca, and P), and amino N contents of all three kimchi groups (*n* = 27, *P* < 0.05). The abundance of Lactobacillaceae family was negatively correlated with most other bacterial families in all three kimchi groups. Moreover, Lactobacillaceae was the only family that correlated positively with the fermentation duration. This indicates the predominance of Lactobacillaceae over the other bacterial taxa.

A negative correlation was found between the abundance of Leuconostocaceae and Fusobacteriaceae, and the Mg content. The abundance of most bacterial families exhibited negative or no significant correlation with amino N contents, except Lactobacillaceae, which positively correlated with amino N contents, and Leuconostocaceae, which correlated positively with the Ca and P contents (Fig. 7).

**Figure 8:**
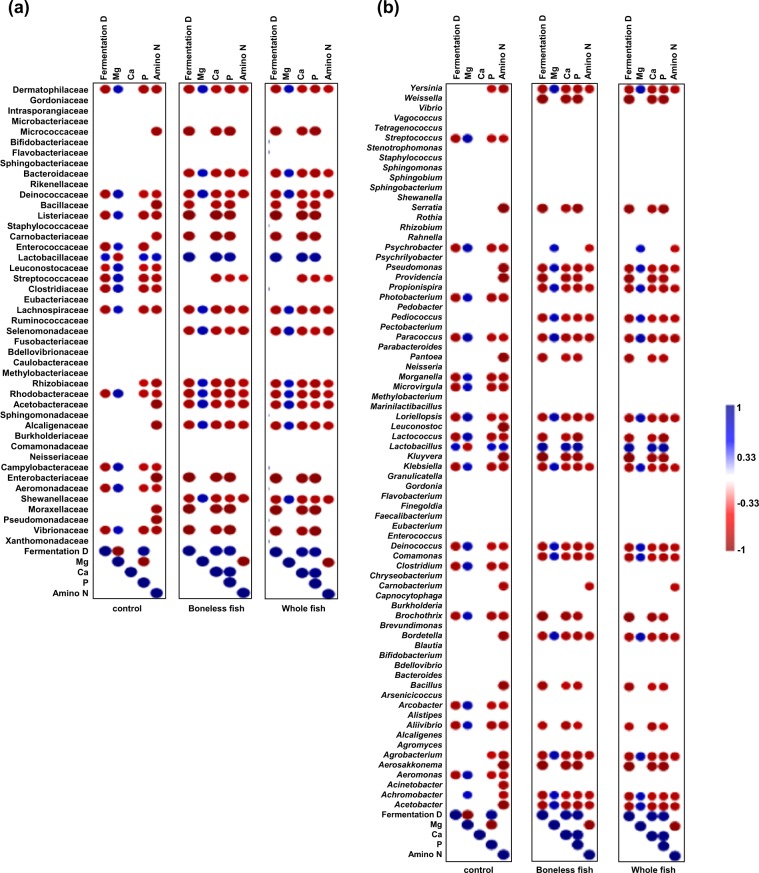
Correlograms representing the matrices of Spearman’s rank order correlation coefficient (r) between the different bacterial taxa at (**a**) family and (**b**) genus levels and the fermentation duration, magnesium (Mg), calcium (Ca), phosphorus (P) inorganic minerals and amino nitrogen (N) contents of kimchi prepared by adding 10% boneless or whole gizzard shad fish slices. The control kimchi group was prepared without adding gizzard shad fish. Only significant (*P* < 0.05) positive (blue) and negative (red) correlations are shown in the graph.

The correlation of the inorganic elements and amino N contents with fermentation duration was analysed. In the control kimchi group, the Mg contents were negatively correlated, and the P contents were positively correlated, with the fermentation duration. In the BLGS and WGS kimchi groups, a positive correlation was found between the fermentation duration and the Ca, and P contents (Fig. 8).

The correlation between the abundance of bacterial taxa and the fermentation duration, Ca, Mg and P and amino N content was also analysed. There was a significant positive correlation (*P* < 0.05) between the abundance of Lactobacillaceae and the fermentation duration in all the groups. However, a significant negative correlation (*P* < 0.05) was only found between the abundance of Leuconostocaceae and the fermentation duration in the control kimchi group. Additionally, there was a negative correlation between the abundance of Lactobacillaceae and the Mg contents in the control kimchi group. Furthermore, a positive correlation was observed between the abundance of Lactobacillaceae and the P and amino N contents in the control kimchi group. In the BLGS and WGS kimchi groups, the abundance of Lactobacillaceae positively correlated with the Ca, and P contents (Fig. 8a). The correlation between the microbes at the genus level and the fermentation duration, inorganic element content, and amino N content between the three kimchi groups was also analysed. The abundance of *Weissella* genus negatively correlated with the fermentation duration in the BLGS and WGS kimchi groups (Fig. 8b). The abundance of *Lactobacillus* genus positively correlated with the fermentation duration in all three kimchi groups. In the control kimchi group, the abundance of *Lactobacillus* genus negatively correlated with the Mg contents, and positively correlated with the P and amino N contents. In the BLGS and WGS kimchi groups, the abundance of *Lactobacillus* positively correlated with the Ca and P contents. The abundance of *Leuconostoc* genus negatively correlated with the amino N content only in the control group and did not exhibit any significant correlation (*P* < 0.05) in the other kimchi groups (Fig. 8b). Correlograms revealing the correlation at other taxonomic levels (phylum, class, and species levels) are shown in Fig. S4.

## Discussion

Understanding the composition of the microbial community in food, including kimchi, is important as these microbes may directly influence the human gut microbiome and health^1,18^. Kimchi ingredients affect the microbial composition during fermentation. Hence, studying the effect of different ingredients will contribute to developing the best recipes that control the microbial fermentation process and enhance the health benefits, shelf life, and sensory properties of the final product. In this study, the effect on the microbial composition of radish kimchi preparations supplemented with fresh seafood, in the form of BLGS and WGS, at three different fermentation time points was investigated. Moreover, the inorganic elements and amino N contents, and their correlation with the kimchi microbial composition, were evaluated.

Generally, seafood products are a good source of protein and inorganic elements. In particular, gizzard shad which can be consumed with its bones can be a good source of Ma, Ca and, P, which are components of the bones. In this study, the Ca, P, and amino N contents increased in the BLGS- and WGS-supplemented kimchi groups compared with the control kimchi group, as the fermentation progressed. Inorganic elements, such as Ca and P, are essential nutrients for bacteria for a variety of cellular metabolic processes. P is a major constituent of biomolecules, such as nucleotides and phospholipids^19^. Ca is also vital for cellular metabolic processes as it has a role in lipid synthesis and membrane composition, in addition to its roles in mediating the function of proteins involved in DNA replication^20^. Therefore, it was proposed that changes in the nutritional profile of the three kimchi groups may also contribute to differential microbial compositions by creating a microecosystem that preferentially enables the growth of certain bacterial species over others.

The difference in the microbial composition of the three kimchi groups was mainly due to the abundance of different families of bacteria; *i.e*. Lactobacillaceae vs. Leuconostocaceae. In the BLGS and WGS kimchi groups, the abundance of *Leuconostoc* genus was higher than that of *Lactobacillus* genus, which was more abundant in the control group, and *Weissella* was the second-most abundant genus. In the control kimchi group, the abundance of *L. sakei* rapidly increased at the optimal- and over-ripening stages. One of the most common species in kimchi is *L. sakei*, which imparts several beneficial characteristics. However, the abundance of *L. sakei* was caused by a drop in pH and is usually linked to rapid acid deterioration of kimchi^20^. The data indicates that adding seafood might contribute to shelf life extension of kimchi by favouring the growth of *Leuconostoc* over *Lactobacillus* at late fermentation stages.

The abundance of the *Leuconostoc* genus is reportedly high during the early stages of fermentation in general. However, as fermentation progresses, the pH reduces and *Lactobacillus* becomes the predominant genus as it is relatively more tolerant to low pH^4,10^. The *Lactobacillus* genus is not only more low pH-tolerant but it also exhibits greater tolerance to higher salinity compared with the *Leuconostoc* genus^21^. *L. rapi* was the predominant species at the optimal- and over-ripening stages in the BLGS kimchi group; while *L. rapi* was the predominant species at the optimal ripening stage, and both *L. sakei* and *L. rapi* were the predominant species at the over-ripening stage in the WGS kimchi group. Although *L. rapi* was not previously reported as a predominant species in kimchi, other related species such as *Leuconostoc mesenteroides* were reported to be predominant in kimchi, especially at the optimal-ripening stage. Metatranscriptomic analysis of kimchi has revealed that the expression of the lactic acid fermentation genes in *L. mesenteroides* is highly active during the early fermentation stages and the corresponding genes in *L. sakei* are highly expressed during later fermentation stages^22^. Earlier reports suggested that *Leuconostoc* and *Weissella* species exhibit antagonistic activity against *L. sakei* by producing non-proteinaceous antibacterial substances. This antagonistic activity is believed to extend the shelf life of kimchi and to improve the flavour at late-ripening stages of fermentation^23^. The *Leuconostoc* genus is known for imparting several health-promoting as well as taste-improving properties to kimchi. The genes encoding mannitol dehydrogenase were found in all the *Leuconostoc* species in kimchi, whereas these genes were absent in *L. sakei*^22^. Mannitol is produced by the reduction of fructose mainly by heterofermentative LAB during the fermentation of vegetables. Additionally, mannitol contributes to the refreshing flavour and other desirable characteristics, such as the production of antioxidants^7,9^. These findings could explain the refreshing flavour of kimchi supplemented with fresh seafood at the optimal- and over-ripening stages, during which there is high abundance of *Leuconostoc*.

In this study, the microbial composition in the three radish kimchi groups was correlated with the inorganic elements and amino N contents. A previous study reported the differential requirement of inorganic minerals for several LAB. The study reported that P was required for the growth of almost all the tested LAB and that although Mg stimulated the growth of certain LAB, it was not essentially required for their growth^24^. In the present study, the Ca and P contents positively correlated with the abundance of Leuconostocaceae, which were high in the BLGS and WGS kimchi groups. A negative correlation was observed between the abundance of *Weissella* and the fermentation duration and the Ca, and P contents in the BLGS and WGS kimchi groups, which could explain the decrease in their abundance as fermentation progresses in these groups (Fig. 8b). Correlation analysis indicated that the difference in the correlation direction between the abundance of bacteria taxa and the inorganic elements and amino N contents between different kimchi groups must be considered for controlled kimchi fermentation. However, it is difficult to generalize the conclusions based on this test, as many other factors could be involved in the fermentation process, such as bacterial utilization or synthesis of certain elements, and the time required for the release of elements from the different ingredients.

To summarize, the present study monitored the changes occurring in radish kimchi microbial composition and inorganic elements and amino N contents upon addition of fresh seafood in the form of BLGS and WGS. The results indicated that there was a marked alteration in the microbial composition of kimchi. The abundance of *L. rapi*, which contributes to improved flavour, was higher in the BLGS and WGS kimchi groups than in the control kimchi group, in which the predominant species was *L. sakei*, which is associated with acidic deterioration of kimchi at the late-ripening stage. Furthermore, adding the whole fish resulted in a better kimchi nutrition profile and equilibrium between the two dominant microbes at the late-ripening stage. The data suggest a beneficial role of adding fish during kimchi fermentation, which is a practice adopted in several Korean coastal provinces. Studying the correlation between bacterial taxa and the different inorganic elements may be useful for controlling fermentation by promoting the growth of certain taxa through adjustments to kimchi ingredients. This study demonstrated that the abundance of certain bacteria during fermentation can be used for customizing kimchi quality and flavour. Further studies are required to understand the kimchi fermentation process in the context of the ecological variables.

## Materials and Methods

### Preparation of radish kimchi samples

Sliced gizzard shads were purchased from the Gijang Fish Market (Busan, Korea) in the size of 3 × 1 cm or less in two type of forms. Half of them did not have bones (BLGS), while the other half did have bones (WGS). Three different groups of radish kimchi batches were prepared using the traditional Korean method: control kimchi, kimchi containing slices of BLGS, and kimchi containing slices of WGS. The ingredients of the control kimchi group were as follows: Korean radish (4000 g); sun-dried salt (200 g); sugar (60 g); glutinous rice flour (200 g); garlic (45 g); ginger (15 g); red pepper powder (200 g); sesame (30 g); anchovy sauce (60 g); and fermented sand lance (60 g). The other two kimchi groups were prepared similarly with a change in the amount of radish (3600 g) and the addition of BLGS or WGS (3 × 1 cm; 400 g).

The kimchi samples were prepared as previously described by *Park et al*.^17^. Briefly, fresh Korean radish was cut into cubes of approximately 4 × 3 × 1.5 cm in size. The cubes were soaked in salt for 1 h and washed twice in running water. The radish cubes were dried thoroughly and mixed with minced garlic and ginger. The other ingredients were added to the mixture in the amounts mentioned above. The prepared kimchi batches underwent fermentation in large containers for 24 h at room temperature. The fermented samples were then stored in the refrigerator at 4 °C. The samples (300 g) from the prepared radish kimchi were collected in sterilized containers at three fermentation time points (initiation, same storage day; optimal-ripening, at 2 weeks; over-ripening, at 6 weeks). The samples were ground using an electric mixer (HMF-3500TG, Hanil Electronic, Seoul, Korea) and filtered using multiple layers of cheese cloth. The filtrates were used for the measuring the inorganic elements and amino N contents and for the metagenomic microbiome analysis.

### Assessment of inorganic elements and amino N

The inorganic elements (Mg, Ca, and P) were quantified in the radish kimchi using an inductively coupled plasma mass spectrometer (ICP-MS, Agilent 7700 series; Agilent Technologies, Santa Clara, USA), following the standard method. Ultrapure water with a resistivity of 18.2 MΩ or higher, and semiconductor-grade nitric acid (HNO_3_) were used (Dongwoo Fine-Chem, Pyeonack, Korea) to conduct this assay. Briefly, 5 g of the sample was dissolved in 50 mL deionized water and centrifuged for 15 min at 10,000 *g* (Avanti JE Centrifuge; Beckman Coupler, Fulleron, USA). The supernatant was used for the quantification of inorganic elements and amino N. Standard solution were prepared of different concentrations (0, 1, 5, 10, 50, 100, 500, and 1000 g/L) for each inorganic element in 1% HNO_3_. The inorganic element content was quantified using the respective calibration curve. The amino N content was determined using the formol titration method as described in AOAC^25^.

### Metagenomic DNA extraction, sequencing, and 16S library preparation

To prepare the radish kimchi for DNA extraction, the ground samples were centrifuged for 15 min at 10,000 *g* at 4 °C to remove excessive water. From the pellet, 250 mg of the sample was subjected to DNA extraction using the PowerSoil^®^ DNA Isolation Kit (MO BIO Laboratories, Carlsbad, USA), following the manufacturer’s instructions. The concentration and quality of the extracted DNA were evaluated using a NanDrop2000 spectrophotometer (Thermo Fisher Scientific, Wilmington, USA) and by agarose gel electrophoresis. The samples were stored at −20 °C in TE buffer until use.

For metagenomic analysis, the 16S rRNA variable regions, V3 and V4 were amplified using the Herculase II fusion DNA polymerase Nextera XT Index Kit V2 in Illumina^®^ MiSeq^®^ platform at Macrogen (Seoul, South Korea). The following primer pairs were used for amplification: (F), 5′-TCGTCGGCAGCGTCAGATGTGTATAAGAGACAGCCTACGGGNGGCWGCAG-3′; (R), 5′GTCTCGTGGGCTCGGAGATGTGTATAAGAGACAGGACTACHVGGGTATCTAATCC-3′.

The resulting paired-end reads were merged using fast length adjustment of short reads (FLASH)^26^. The raw reads were then purified and trimmed to remove the low quality and short reads, and the adaptors. The CD-HIT-OTU-MiSeq and UCLUST algorithm were used for clustering and annotation of the qualified sequences into the respective operational taxonomic units (OTUs) at 97% cut off against the Greengenes database^27–29^. Diversity statistics, taxonomic alignment, and microbiome analysis were performed using the quantitative insights into microbial ecology version 2 (QIIME2) pipeline^30^. The obtained sequences were deposited in the sequence read archive (SRA) of the National Center for Biotechnology Information database under the BioProject ID PRJNA534409.

### Statistical analysis

The alpha-diversity and beta-diversity analyses were performed using the QIIME2 scripts and R software (version 3.1.3). The principle coordinate analysis (PCoA) was estimated based on the Euclidean, Bray-Curtis, and weighted UniFrac distances. All statistical analyses were conducted after testing the data for normality. Analysis of variance (ANOVA) followed by least significant difference (LSD) test was used for normally distributed data. For data which did not follow normal distribution, non-parametric Kruskal–Wallis test was performed using the Statistical Analysis Systems (SAS Institute, Cary, USA). The difference was considered statistically significant when *P* value was less than 0.05. Bacterial taxa at different taxonomic levels in the three radish kimchi groups were tested for correlation with the fermentation duration, inorganic elements and amino N contents using Spearman’s rank correlation. Correlation analysis and correlograms representing the Spearman’s rank correlation coefficient (r) matrices were performed using the PAleontological STatistics software package (PAST) version 3.23^31^.

## Supplementary information

Supplementary information
