# Supplementary material for: Effect of Seafood (Gizzard Shad) Supplementation on the Chemical Composition and Microbial Dynamics of Radish Kimchi during Fermentation
Source: Sci Rep. 2019 Nov 27;9:17693. doi: 10.1038/s41598-019-54318-4 (PMC6881437; doi:10.1038/s41598-019-54318-4)
Supplement: Supplementary file 1 — Supplementary information [file 41598_2019_54318_MOESM1_ESM.pdf]

Supplementary Information

Effect of Seafood (Gizzard Shad) Supplementation on the Chemical Composition and Microbial Dynamics of Radish Kimchi during Fermentation

Mohamed Mannaa[1,2], Young-Su Seo[1,*], Inmyoung Park[3,*]

[1]Department of Integrated Biological Science, Pusan National University, Busan 46241, Korea

[2]Department of Plant Pathology, Cairo University, Giza 12613, Egypt

[3]Department of Oriental Food and Culinary Arts, Youngsan University, Busan 48015, Korea

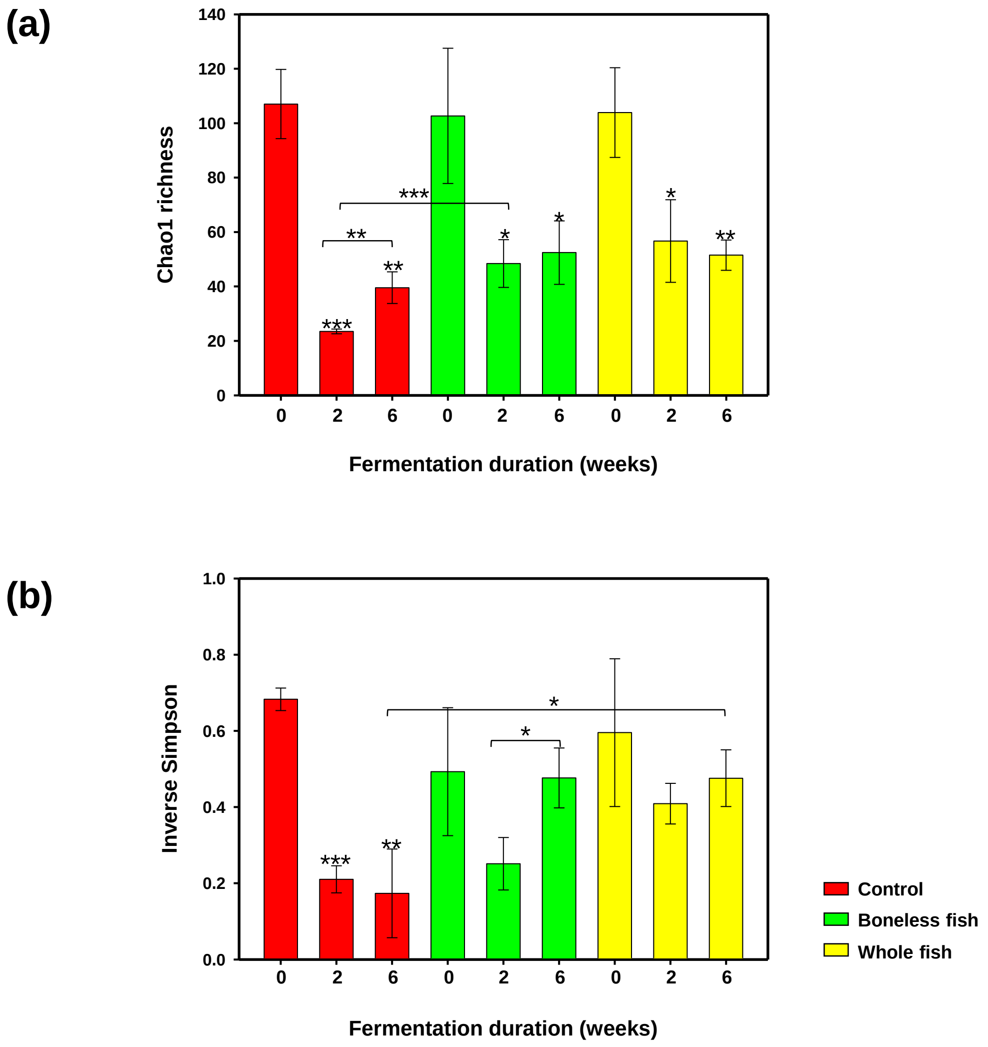

Figure S1 Alpha diversity of radish kimchi microbiota prepared by adding 10% boneless or whole gizzard shad fish slices over different fermentation durations. The control kimchi group was prepared without adding gizzard shad fish. (a) Chao1 diversity index and (b) Inverse Simpson's index. Data are represented as the mean ± standard deviation for each kimchi group. *** $P < 0.001$, ** $P < 0.01$, and * $P < 0.05$.

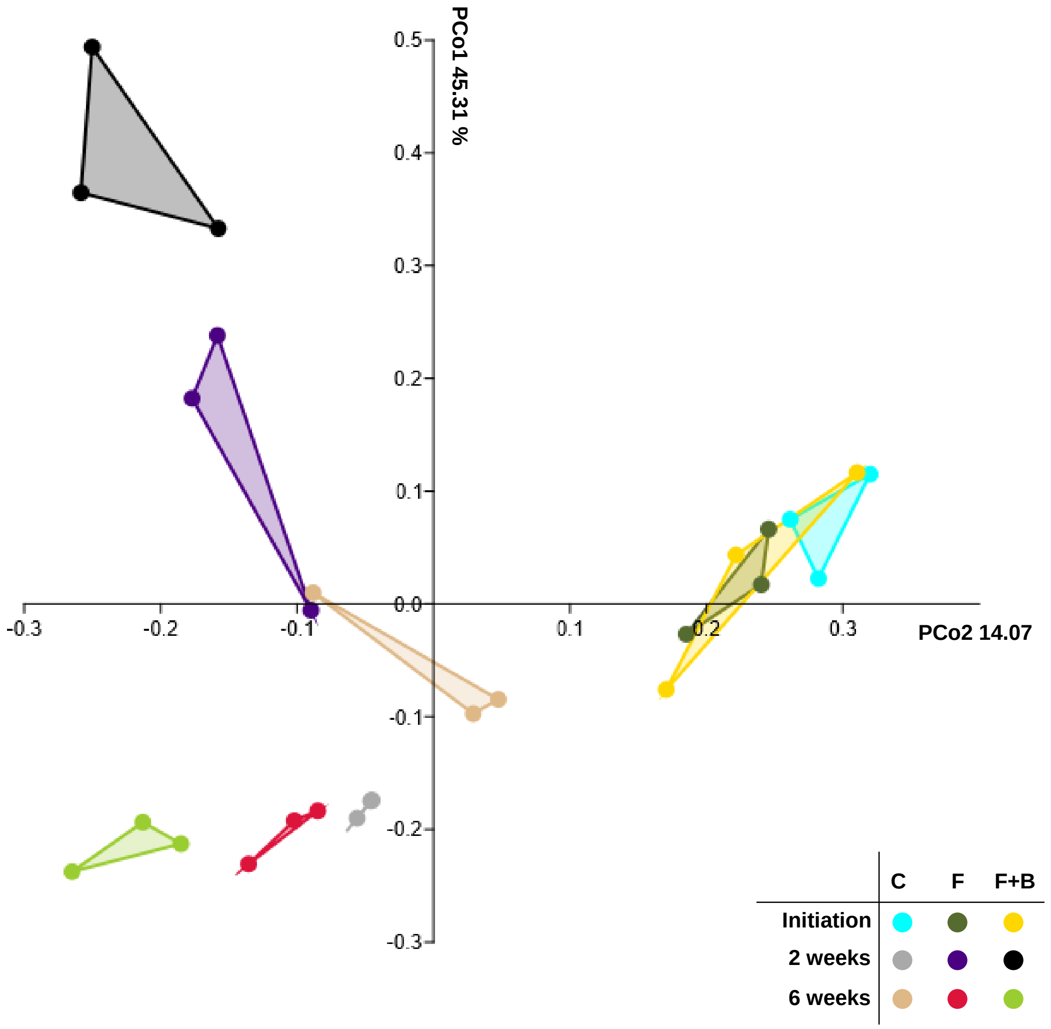

Figure S2 Principle coordinate analysis (PCoA) of the Euclidean distance representing the beta-diversity in the microbiota of radish kimchi prepared by adding 10 % boneless or whole gizzard shad fish slices over different fermentation time points. Each dot represents a replicate of the different groups of kimchi prepared by adding 10 % boneless or whole gizzard shad fish slices over different fermentation time points (initiation, 0 weeks; optimal ripening, 2 weeks; over ripening, 6 weeks). The control kimchi group was prepared without adding gizzard shad fish. Clustering was clear between the kimchi groups at the initiation of the fermentation and at the optimum-ripening and the over-ripening stages.

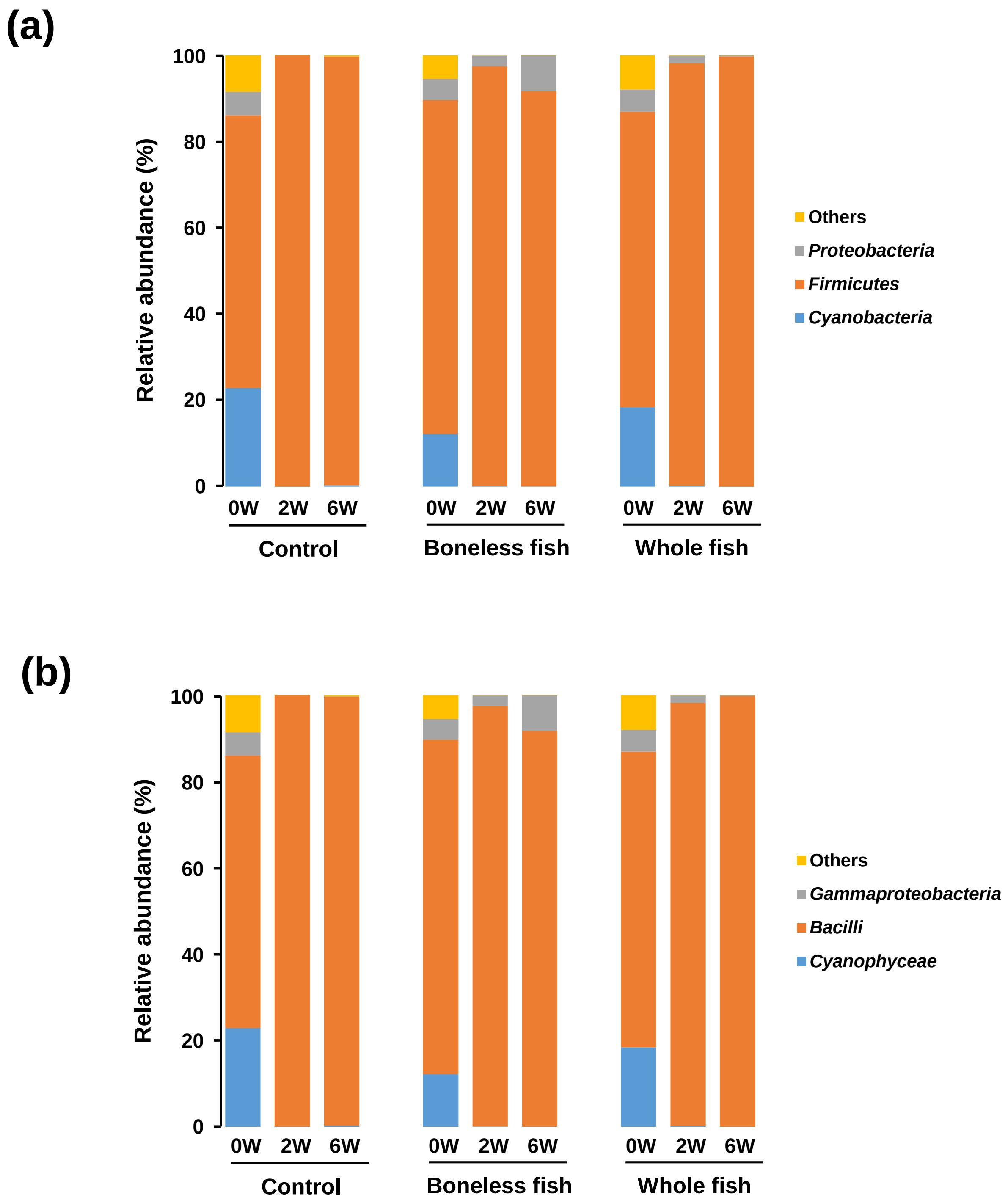

Figure S3 Stacked bar graphs representing the microbiome composition of radish kimchi prepared by adding 10% boneless or whole gizzard shad fish slices over different fermentation durations. The microbial composition at the (a) phylum level and (b) class level.

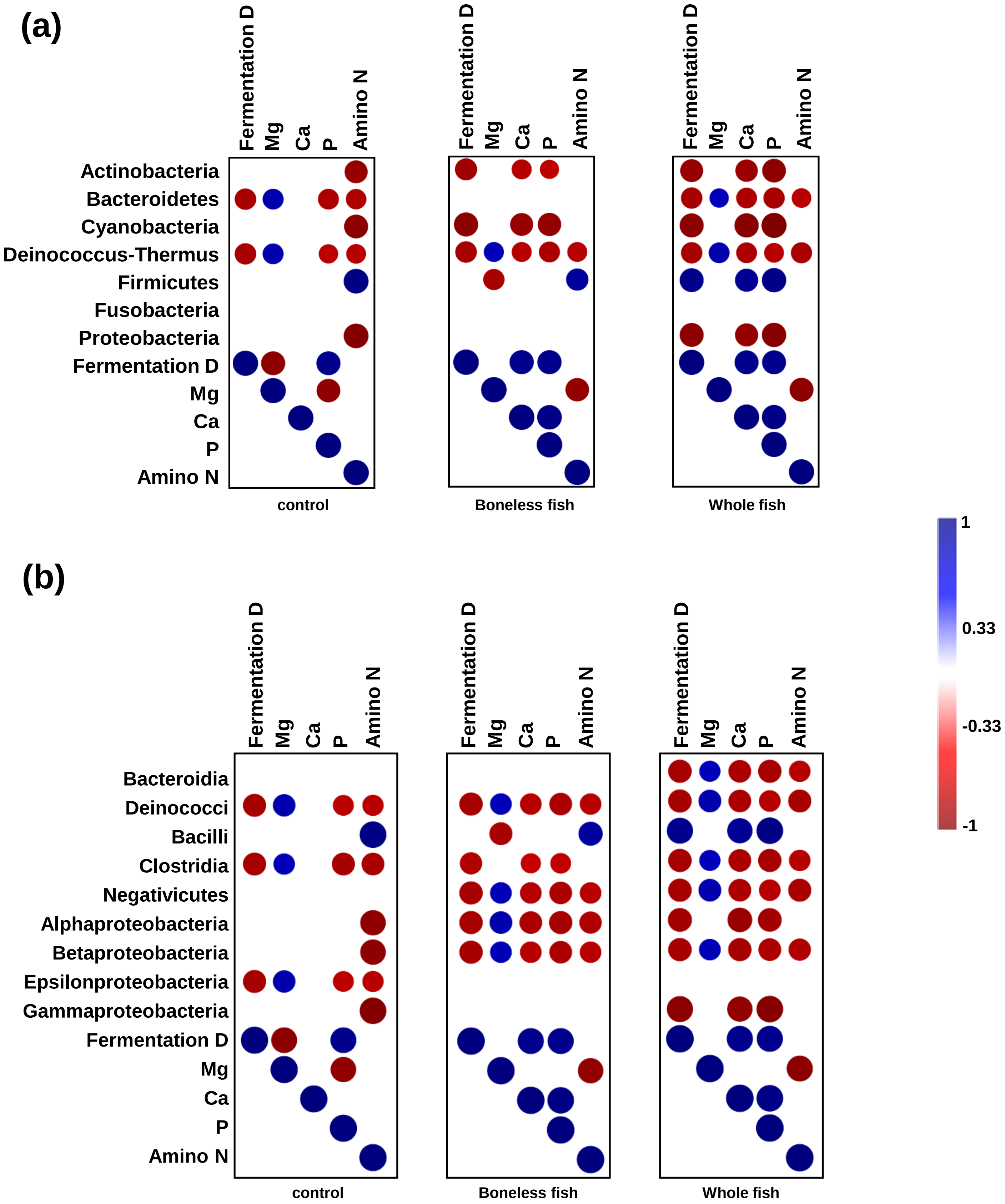
(a)
Fermentation D
Mg
Ca
P
Amino N
Actinobacteria
Bacteroidetes
Cyanobacteria
Deinococcus-Thermus
Firmicutes
Fusobacteria
Proteobacteria
Fermentation D
Mg
Ca
P
Amino N
control
Boneless fish
Whole fish
(b)
Bacteroidia
Deinococci
Bacilli
Clostridia
Negativicutes
Alphaproteobacteria
Betaproteobacteria
Epsilonproteobacteria
Gammaproteobacteria
1
0.33
-0.33
-1

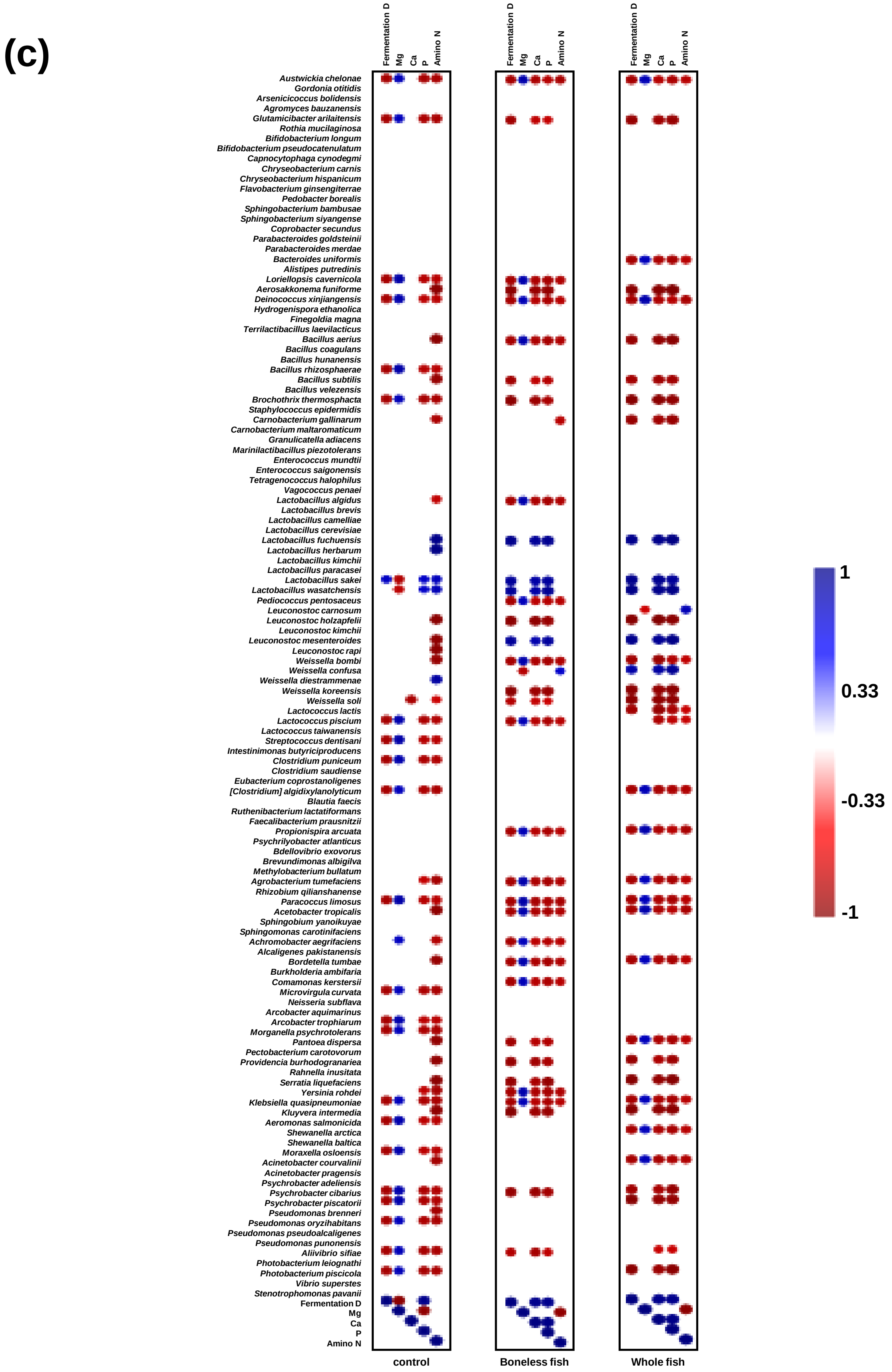

Figure S4 Correlograms representing the matrices of Spearman's rank order correlation coefficient (r) between different bacterial taxa at (a) phylum, (b) class and (c) species levels and different fermentation durations, the contents magnesium (Mg), calcium (Ca), phosphorus (P) and the content of amino nitrogen (N). Radish kimchi was prepared by adding 10 % boneless or whole gizzard shad fish slices. The control kimchi group was prepared without adding gizzard shad fish. Only significant ($P < 0.05$) positive (blue) and negative (red) correlations are shown in the graph.
